# Genomics Analysis and Nomogram Risk Prediction of Occult Lymph Node Metastasis in Non-Predominant Micropapillary Component of Lung Adenocarcinoma Measuring ≤ 3 cm

**DOI:** 10.3389/fonc.2022.945997

**Published:** 2022-07-13

**Authors:** Kun Wang, Mengchao Xue, Jianhao Qiu, Ling Liu, Yueyao Wang, Rongyang Li, Chenghao Qu, Weiming Yue, Hui Tian

**Affiliations:** ^1^ Department of Thoracic Surgery, Qilu Hospital of Shandong University, Jinan, China; ^2^ Department of Pathology, Qilu Hospital of Shandong University, Jinan, China

**Keywords:** occult lymph node metastasis, adenocarcinoma, micropapillary, EGFR 19del, nomogram

## Abstract

**Background:**

The efficacy of sublobar resection and selective lymph node dissection is gradually being accepted by thoracic surgeons for patients within early-stage non-small cell lung cancer (NSCLC). Nevertheless, there are still some NSCLC patients develop lymphatic metastasis at clinical T1 stage. Lung adenocarcinoma with a micropapillary (MP) component poses a higher risk of lymph node metastasis and recurrence even when the MP component is not predominant. Our study aimed to explore the genetic features and occult lymph node metastasis (OLNM) risk factors in patients with a non-predominant micropapillary component (NP-MPC) in a large of patient’s cohort with surgically resected lung adenocarcinoma.

**Methods:**

Between January 2019 and December 2021, 6418 patients who underwent complete resection for primary lung adenocarcinoma at the Qilu Hospital of Shandong University. In our study, 442 patients diagnosed with lung adenocarcinoma with NP-MPC with a tumor size ≤3 cm were included. Genetic alterations were analyzed using amplification refractory mutation system-polymerase chain reaction (ARMS-PCR). Abnormal protein expression of gene mutations was validated using immunohistochemistry. A nomogram risk model based on clinicopathological parameters was developed to predict OLNM. This model was invalidated using the calibration plot and concordance index.

**Results:**

In our retrospective cohort, the incidence rate of the micropapillary component was 11.17%, and OLNM was observed in 20.13% of the patients in our study. ARMS-PCR suggested that EGFR exon 19 del was the most frequent alteration in NP-MCP patients compared with other gene mutations (frequency: 21.2%, P<0.001). Patients harboring exon 19 del showed significantly higher risk of OLNM (P< 0.001). A nomogram was developed based on five risk parameters, which showed good calibration and reliable discrimination ability (C-index = 0.84) for evaluating OLNM risk.

**Conclusions.:**

Intense expression of EGFR exon 19 del characterizes lung adenocarcinoma in patients with NP-MCP and it’s a potential risk factor for OLNM. We firstly established a nomogram based on age, CYFRA21-1 level, tumor size, micropapillary and solid composition, that was effective in predicting OLNM among NP-MCP of lung adenocarcinoma measuring ≤ 3 cm.

## Introduction

Lung cancer is the second most prevalent tumor and remains as the major cause of malignancy-related mortality globally according to the latest report (1). With the increasing popularity of low-dose spiral computed tomography (CT) screening, the incidence of early clinical T stage lung malignancy has been increasing (2). As the number of patients with small ground glass nodule increases, more thoracic surgeons have accepted segmental or subsegmental resection and selective lymph node dissection as optimized treatment modalities (3, 4). However, there are still some patients with early clinical stage occur lymph node metastasis. And part of the cases is characterized by occult lymph node metastasis (OLNM). Therefore, for these patients with potential OLNM risks, lobectomy and systematic lymphadenectomy is more valuable for prolonging patient survival.

Lung adenocarcinoma, as the most common pathological subtype of lung cancer, presents with diverse histological patterns and molecular features (5). The World Health Organization classifies invasive adenocarcinoma (IAC) into five histologic categories: acinar, lepidic, mucinous, papillary, solid, and micropapillary. And it’s reported that the presence of micropapillary structures often has a relationship with vascular or lymphatic invasion, leading to poor prognosis (6–8). In fact, the incidence of non-predominant micropapillary patterns (NP-MCP) is more common than micropapillary predominant adenocarcinoma (9). However, specific genetic variants and mechanisms of OLNM have not been clearly described. And a risk prediction model of OLNM in lung adenocarcinoma patients with NP-MCP needs to be developed.

In our study, we explored the risk factors for OLNM in a large patient’s cohort diagnosed with lung adenocarcinoma with NP-MPC. We also compared the genetic alterations in NP-MPC patients and identified the genetic traits responsible for OLNM. On the basis of the clinicopathological parameters, a nomogram model for predicting preoperative OLNM risk was developed.

## Materials and Methods

### Patients

**Figure 1:**
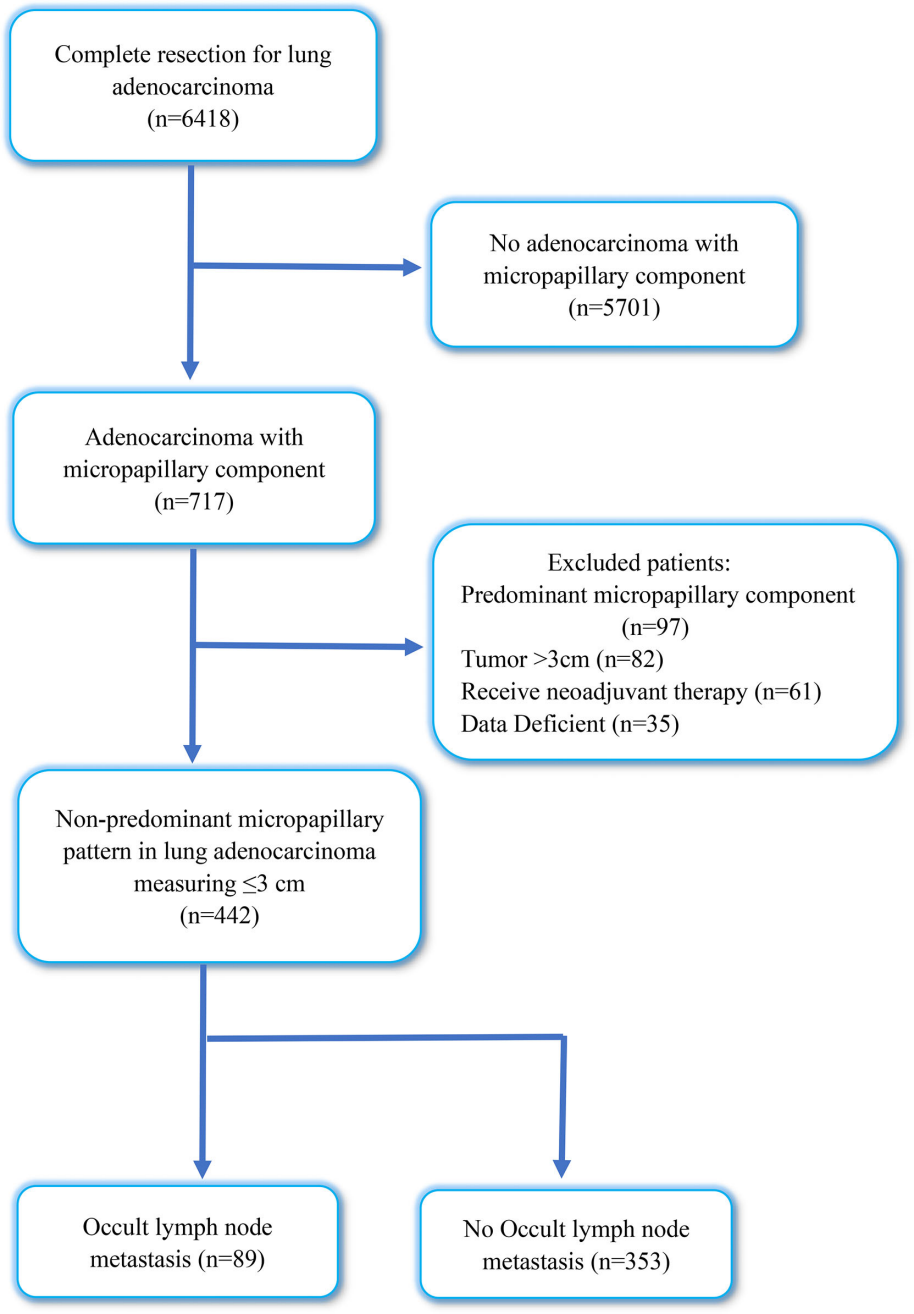
Flow diagram of patients excluded in this research. OLNM, occult lymph node metastasis; NOLNM, non- occult lymph node metastasis.

This study included 6418 patients with IAC who underwent lobectomy plus systematic lymph node dissection at the Qilu Hospital of Shandong University between January 2019 and December 2021. We excluded patients with non-adenocarcinoma with micropapillary component (n = 5701), lung adenocarcinoma with predominant micropapillary components (n = 97), tumor diameter >3 cm (n = 82), preoperative neoadjuvant therapy (n = 61), and incomplete medical records (n = 35). After this exclusion, our study enrolled 442 patients with tumor size ≤ 3 cm, diagnosed with IAC with NP-MCP. **Figure 1** shows a flowchart of the included patients. This study was approved by the Institutional Review Board of the Qilu Hospital of Shandong University (KYLL-202008-023-1) for the use surgical samples and patient data analysis. A written informed consent was signed by each patient.

### Clinicopathological Data Evaluation

Clinicopathological data were collected from the patient medical record management system, including sex, age, smoking history, serum tumor marker levels (including CEA, CYFRA21-1, CA125, SCC, pro-GRP and NSE), preoperative test results, tumor size and location, routine pathological reports, and pathological tumor-node-metastasis (TNM) stage. Preoperative assessment of lymph node metastasis was evaluated by experienced diagnostic radiologist using CT or ^18^F-FDG-PET-CT. OLNM was defined as the absence of positive signs of lymph node metastasis found during preoperative examination (CT or PET-CT), lymph node metastasis was confirmed with postoperative pathological results (10).

According to standard pathological section preparation procedures, surgical lung tissues were removed, fixed with formalin, sectioned, and stained with hematoxylin and eosin. Two experienced pathologists determined the patient’s diagnosis, were blinded to the patient data, and independently evaluated each tissue section. According to the 4th version of the WHO Lung Tumors Classification, histological subtypes were classified (11). We identified invasive adenocarcinoma (IAC) and excluded adenocarcinoma in situ, minimally invasive adenocarcinoma, and invasive mucinous adenocarcinoma. The histopathological subtypes of IAC including lepidic adenocarcinoma, acinar adenocarcinoma, papillary adenocarcinoma, micropapillary adenocarcinoma and solid adenocarcinoma. The International Association for the Study of Lung Cancer (IASLC) eighth TNM classification was used for staging (12). The component with the largest proportion was described as the predominant component. The non-predominant component was defined as a subtype occupying no less than 5% but was not predominant.

### DNA Purification and Quantification

All formalin-fixed paraffin-embedded (FFPE) specimens were cut to 5-8 µm thickness, Thereafter, 2–5 sections of tissues with at least 30% tumor cells were used for DNA and RNA extraction using the FFPE DNA/RNA nucleic acid extraction kit (Item No: 8.0223601X036G, Amoy Diagnostics Co, Xiamen, China). After separating DNA and RNA, the concentration of DNA and RNA was determined using a microspectrophotometer. RNA concentrations ranged from 10 to 500 ng/μL and DNA concentrations were >2 ng/μL.

### Genomics Mutation Analysis

Twelve PCR tube gene mutation detection kits (Item No: 8.0126301W006A, Amoy Diagnostics Co, Xiamen, China) were used to detect nine targeted gene alterations (including EGFR 18-20 exon, KRAS 2 exon, BRAF 15 exon, HER2 20exon, NRAS 3 exon, PIK3CA 20/9 exon, ALK fusion gene, ROS1 fusion gene and RET fusion gene). A MET 14 exon jump mutation detection kit (Item No: 8.0126101X024H, Amoy Diagnostics Co, Xiamen, China) was used to detect MET 14 exon jump mutation. The reaction mixture was prepared in accordance with these instructions. PCR was performed using Stratagene Mx3000P™ system (Amoy Diagnostics Co, Xiamen, China). Thermal cycling conditions were as follows: 1 cycle at 42°C for 5 minutes and 95° C for 5 minutes; followed by 10 cycles of 95°C for 25 seconds, 64°C for 20 seconds, and 72°C for 20 seconds; and 36 cycles of 93°C for 25 seconds, 60°C for 35 seconds, and 72°C for 20 seconds. To ensure the reliability of the detection results, we adopted the multiple quality control principle, which included negative and positive control groups + internal quality control + external quality control.

### Immunohistochemistry Validation in Resected Patients

Genetic variants significantly expressed in NP-MCP patients were detected using ARSM-PCR. Then the protein expression of the ARSM-PCR-detected variant was validated using IHC. Rabbit anti-EGFR polyclonal antibody (Abcam, Cambridge, MA, USA), anti-KRAS antibody (Thermo Fisher Scientific, Rockford, IL, USA), rabbit monoclonal ALK antibody (Cell Signaling Technology, Danvers, MA, USA), anti-ROS1 rabbit monoclonal antibody (Cell Signaling Technology, Danvers, MA, USA), RET antibody (EPR2871, 1:250) (Epitomics, Inc., Burlingame, CA), and HER2 antibody (Ventana Medical Systems, Tucson, AZ, USA) were used for immunohistochemistry analysis.

### Construction and Evaluation of the Nomogram

To develop a practical method for predicting the OLNM risk in patients with early-stage lung cancer, we established a nomogram model using the independent clinicopathological risk indicators found in the logistic regression analysis. The receiver operating characteristic (ROC) curve was calculated to evaluate the predictive ability. Calibration plots were performed to evaluate the model compliance.

### Statistical Analyses

The mean and standard deviation were utilized to represent the continuous variables subject to normal distribution, and the mean values were compared using a t-test. The non-normally distributed variables are represented by a median and inter-quartile range, and Mann-Whitney U test was performed to compare the distributions among subgroups. Categorical variables were displayed as ratios, and variables among different groups were compared using Fisher’s exact test and χ2 test. The risk variables of OLNM patients with NP-MCP were investigated using univariate and multivariate logistic regression analyses. Odds ratios (OR) and 95% confidence interval (CI) were calculated. P-value < 0.05 was considered statistically significant. SPSS Statistics software (version 26.0) was utilized to conduct statistical analyses. The R software (version 4.1.0) was used to construct and evaluate the nomogram model.

## Results

### Patient Characteristics

From January 2019 to December 2021, 6418 patients who underwent complete resection for primary lung adenocarcinoma were reviewed in our cohort; IAC with micropapillary component (MPC) was observed in 717 patients, with an incidence rate of MPC of 11.17%. Through inclusion and exclusion screening, a total of 442 patients diagnosed with IAC with NP-MPC were included in this retrospective research, and the incidence rate of NP-MPC was 6.9%. Among them, the overall incidence rate of OLNM was 20.13% (89/442). In our cohort, 204 patients were men (46.2%), and 238 were women (53.8%). The median age of patient was 61 years (27–86 years). A total of 132 patients (29.9%) were current or former smokers, and 310 patients (70.1%) had never smoked. The tumor size ranged from 0.5 to 3 cm (median 2 cm). For TNM stage, the numbers of patients with IA1, IA2, IA3, IB, IIB, IIIA, IIIB and IVA were 20 (4.5%), 152 (34.4%), 82 (18.6%), 64 (14.5%), 67 (15.2%), 51 (11.5%), 1 (0.2%) and 5 (1.1%), respectively.

### Correlation Between Clinicopathological Parameters and Occult Lymph Node Metastasis

Through univariate logistic regression analysis, the clinicopathological parameter characteristics of the different subgroups of OLNM are shown in **Table 1**. We found that age (P = 0.018), CYRRA21-1 level(P < 0.001), tumor size (P = 0.001), resected lymph node number (P < 0.001), TNM stage (P < 0.001), lepidic component (P = 0.001), micropapillary component (P < 0.001) and solid component (P < 0.001) were risk factors for OLNM. Multivariate logistic regression analysis was performed to analyze the correlation between OLNM and clinicopathological variables identified by univariate logistic regression in IAC patients with NP-MCP. Our data showed that age (OR = 0.96; 95% CI: 0.93-0.99; P =0.006), tumor size (OR = 1.89; 95% CI: 1.26–2.97; P =0.005), CYFRA21-1 level (OR = 1.66; 95% CI: 1.39–2.00; P <0.001), micropapillary component (OR = 1.03; 95% CI: 1.01–1.05; P =0.001) and solid component (OR = 1.04; 95% CI: 1.04–1.07; P =0.001) were independent risk factors for OLNM in non-predominant micropapillary component patients (**Table 2**).

**Table 1:** Clinicopathological variables in the cohort of enrolled patients.

Clinicopathological profiles	Total	pN+	pN-	*P* value
Sex				0.241
Male	204	46 (22.5%)	158 (77.5%)	
Female	238	43 (18.1%)	195 (81.9%)	
Age, years	61 (54-66)	57 (52-65)	62 (55-67)	**0.018**
Smoking history				0.682
Non-smoker	310	64 (20.6%)	246 (79.4%)	
Former/Current smoker	132	25 (18.9%)	107 (81.1%)	
CEA, ng/ml	3.19 (1.92-6.06)	4.02 (2.05-8.18)	3.15 (1.87-5.86)	0.078
CYFRA21-1, ng/ml	2.22 (1.67-3.10)	3.43 (2.29-4.18)	2.10 (1.58-2.72)	**<0.001**
SCC, ng/ml	0.80 (0.60-1.13)	0.80 (0.60-1.06)	0.84 (0.60-1.13)	0.957
pro-GRP, pg/ml	39.10 (31.28-47.11)	38.90 (31.74-49.43)	39.10 (30.92-46.71)	0.767
CA125, U/ml	10.90 (7.78-15.23)	12.75 (8.38-18.35)	10.35 (7.53-14.80)	0.057
NSE, ng/ml	17.80 (14.50-21.80)	18.90 (15.28-23.05)	17.70 (14.4-21.56)	0.102
Tumor size, cm	2.00 (1.50-2.70)	2.50 (2.0-3.0)	2.0 (1.5-2.6)	**0.001**
Tumor location				0.202
RUL	140	27 (19.3%)	113 (80.7%)	
RML	49	12 (24.5%)	37 (75.5%)	
RLL	86	11 (12.8%)	75 (87.2%)	
LUL	90	18 (20.0%)	72 (80.0%)	
LLL	77	21 (27.3%)	56 (72.7%)	
TNM stage				**<0.001**
IA1-3	254	1 (1.2%)	253 (98.8%)	
IB	64	0	64 (100%)	
IIB	67	59 (88.1%)	8 (11.9%)	
IIIA	51	27 (52.9%)	24 (47.1%)	
IIIB	1	0	1 (100%)	
IVA	5	2 (40.0%)	3 (60.0%)	
Resected lymph node number, median(IQR)	9 (5-12)	10 (8-14)	8(5-12)	**<0.001**
Acinar component, %	45 (30-70)	55 (30-70)	45 (30-70)	0.348
Lepidic component, %	10 (0-25)	0 (0-10)	10 (0-27)	**0.001**
Papillary component, %	15 (0-30)	15 (0-30)	15 (0-40)	0.076
Micropapillary component, %	10 (5-15)	10 (5-20)	5 (5-10)	**<0.001**
Solid component, %	0 (0-0)	5 (0-10)	0 (0-0)	**<0.001**

CEA, Carcinoembryonic antigen; CYFRA21-1, cytokeratin fragment antigen 21-1; SCC, Squamous cell carcinoma antigen; pro-GRP: progastrin-releasing peptide; CA125, antigen carbohydrate 125; NSE, neuron specific enolase; RUL, Right upper lobe; RML, Right middle lobe; RLL, Right lower lobe; LUL, Left upper lobe; LLL, Left lower lobe; IA, Invasive adenocarcinoma; IQR, interquartile range.Bold represents a p-value <0.05 among subgroups.

**Table 2:** Univariate logistic regression analysis of the OLNM risk.

Characteristics	OR	95% CI	*P value*
Sex			
Male	0.73	(0.43-1. 27)	0.27
Female			
Age, years	0.96	(0.93-0.99)	**0.006**
Tumor size, cm	1.89	(1.26-2.97)	**0.005**
CYFRA21-1, ng/ml	1.66	((1.39-2.00)	**<0.001**
Resected lymph node number, median(IQR)	1.04	(0.99-1.09)	0.73
Micropapillary component, %	1.03	(1.01-1.05)	**0.001**
Solid component, %	1.04	(1.04-1.07)	**0.001**

OLNM, occult lymph node metastasis; CYFRA21-1, cytokeratin fragment antigen 21-1; IQR, interquartile range. Bold represented a p-value < 0.05 among subgroups.

### Frequency of Ten Targeted Gene Alterations

**Figure 2:**
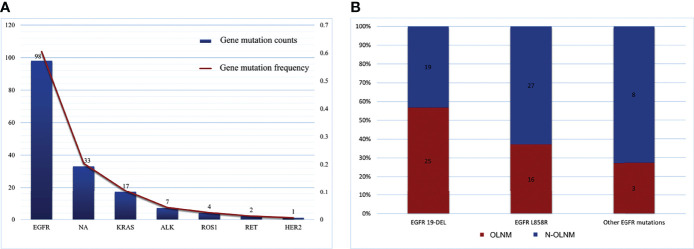
Targeted genetic features detected by ARMS-PCR. **(A)** Gene mutation counts and frequencies of EGFR, KRAS, ALK, ROS1, and HER2; **(B)** Different distributions of EGFR exon mutations in OLNM and NOLNM groups. ARMS-PCR, amplification refractory mutation system-polymerase chain reaction; NA, no genetic mutations detected. OLNM, occult lymph node metastasis; NOLNM, non- occult lymph node metastasis.

Among the 442 patients, 162 underwent gene alteration analysis using ARMS-PCR. Among them, 129 (80.7%) samples were detected to have genetic mutations, and two (1.2%) patients exhibited two mutated driver genes. Ten targeted genetic features are shown in **Figure 2A**. EGFR, KRAS, ALK, RET, ROS1, and HER2 gene mutation frequencies were 60.5% (98/162), 10.5% (17/162), 4.3% (7/162), 1.2% (2/162) 2.5% (4/162), and 0.6% (1/162), respectively. PIK3CA, BRAF, MET, and NRAS gene alterations were not detected in our samples. Moreover, EGFR exon 18 G719X+ exon 20 S768I and EGFR exon 21 L858R+ exon 20 S768I showed synchronous gene alterations in two (1.2%) patients.

### EGFR Mutation Status and Association With Occult Lymph Node Metastasis

EGFR mutations were the most frequent alteration type and were found in 89.9% (44/49) of all OLNM patients. Two samples were identified to have double mutations involving different EGFR exons (EGFR exon 18 G719X+ exon 20 S768I and EGFR exon 21 L858R+ exon 20 S768I). Overall, 27.1% (44/162) of patients had an exon 19 del mutation, followed by exon 21 L858R in 26.0% (42/162) of the patients. And the remaining alteration types with frequencies less than 5.0% were exon 20 20-ins, exon 18 G719X, and exon 20 S768I, with frequencies of 1.9% (3/162), 4.3% (7/162), and 1.2% (2/162), respectively. Furthermore, we identified a statistical correlation between OLNM and EGFR mutations in IAC patients through univariate logistic regression analysis (P <0.001). To further explore the correlation between EGFR exon alterations and OLNM, we performed a subgroup analysis. **Figure 2B** shows the different distributions of EGFR exon mutations between the OLNM and non-OLNM groups. EGFR exon 19 del (25, 56.9%) was the most frequent alteration in OLNM patients, followed by EGFR exon 21 L858R (16, 36.4%) and, other EGFR exon mutations (including exon 18 G719X, exon 20 20-ins, and exon 20 S768I; 3, 6.8%). **Supplementary Figure 1** shows the protein expression of the ARSM-PCR-detected variants, which validated by IHC.

Moreover, univariate analysis (**Table 3**) revealed that lung adenocarcinoma patients with NP-MPC demonstrated a greater incidence of EGFR mutation (P < 0. 001). And the EGFR exon 19 del mutation showed a significantly relationship with OLNM (P < 0.001) compared to EGFR exon 21 L858R (P = 0.176) and other EGFR exon mutations (P = 0.212).

**Table 3:** Comparison of the correlation between EGFR exon mutation and OLNM.

	pN+	pN-	χ^2^	*P value*
EGFR mutation, %				
Negative	5 (10.2%)	59 (52.2%)		
positive	44 (89.8%)	54 (47.8%)	25.238	**<0.001**
Deletion in exon19	25	19	8.773	**0.003**
L858R	16	27	1.831	0.176
Other mutations	3	8	1.556	0.212

OLNM, occult lymph node metastasis; Bold represented a p-value < 0.05 among subgroups.

### Construction and Evaluation of the Nomogram

**Figure 3:**
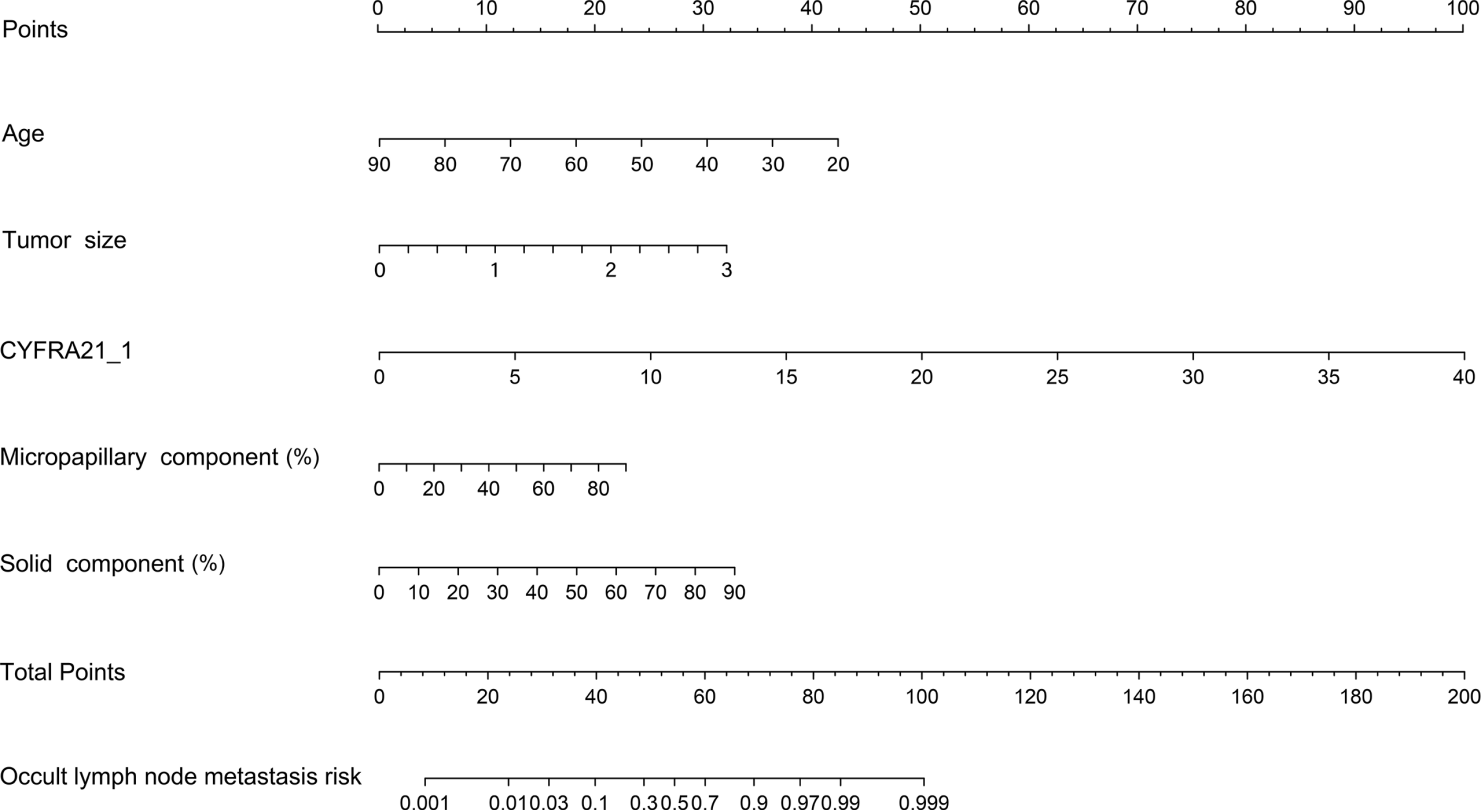
Nomogram model predicting the occult lymph node metastasis risk in stage 1a-c patients with lung adenocarcinoma. CYFRA21-1, cytokeratin fragment antigen 21-1.

**Figure 4:**
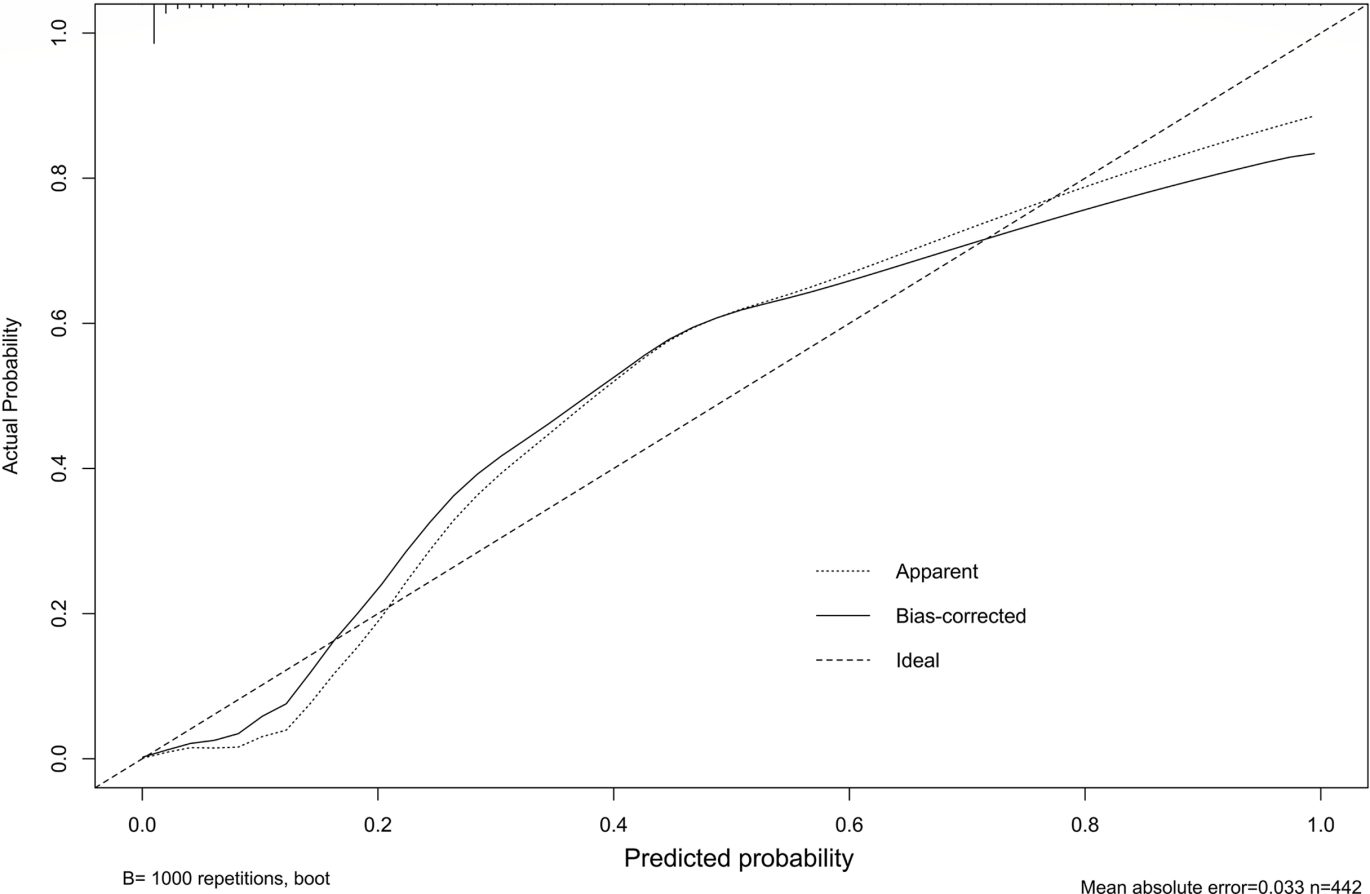
Nomogram prediction model is evaluated by calibration plot.

**Figure 5:**
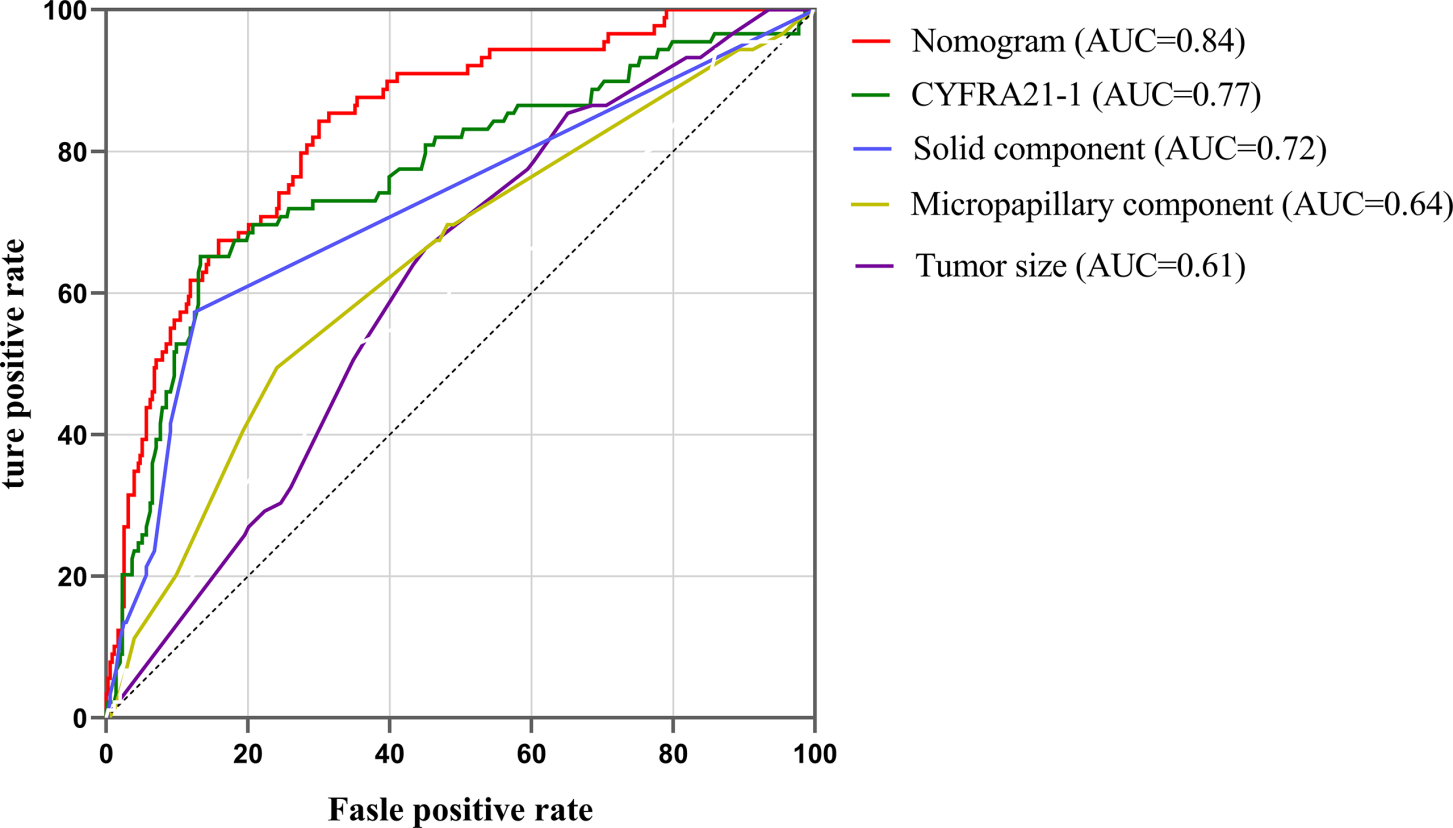
ROC curves of nomogram, CYFRA21-1, solid component, micropapillary component and tumor sizes. ROC, Receiver operating characteristic; AUC, area under the curve.

Our study established a nomogram using the significant factors, including age, tumor size, CTFRA21-1, micropapillary component and solid component, estimated using multivariate logistic regression (**Figure 3**). The total nomogram score can be calculated based on individual scores from all predictive indicators. The potential OLNM risk in patients with NP-MPC in lung adenocarcinoma measuring ≤3 cm can be estimated through calculating the nomogram score. **Figure 4** shows that the actual curve and prediction curve present good concordance, demonstrating stability in predicting OLNM risk. To further verify the reliability of the nomogram model, we evaluated the predictive performance of the nomogram for OLNM and the capacities of these four selected risk indicators. The ROC curves demonstrated the nomogram’s good discriminating accuracy, with an AUC of 0.84 (95% CI: 0.79-0.88), compared to the AUC of 0.77 (95% CI: 0.71-0.83), 0.72 (95% CI: 0.66-0.79), 0.64 (95% CI: 0.58-0.71) and 0.61 (95% CI: 0.56-0.68) for the CYFRA21-1 level, solid component, micropapillary component and tumor size, respectively (all P<0.001; **Figure 5**).

## Discussion

The present study found that age, tumor T stage, CYFRA21-1 level, solid component, and micropapillary component were independent risk factors for OLNM. Targeted genetic analysis showed that EGFR exon 19 del was the most frequent alteration in NP-MCP (21.2%). Furthermore, patients harboring exon 19 del showed a significantly higher risk of OLNM (P < 0.001). Then, a nomogram model was developed to evaluate OLNM risk, which demonstrated consistent discriminating performance (C-index = 0.84) and satisfactory calibration.

Although anatomic lobectomy and systematic hilar mediastinal lymph node dissection has been regarded as a standard therapy for surgically resectable malignant tumor patients, several studies have reported that limited wedge resection or segmentectomy combined with selective lymph node separation may be a more appropriate option for lung cancer patients whose imaging is characterized by small and pure ground glass nodules (13–15). Although patients with an adenocarcinoma tumor lesion diameter ≤ 3cm have a favorable prognosis, OLNM could still develop in a few individuals. Recent researches indicate that OLNM is prevalent in individuals with IAC, especially in patients with peripheral solid NSCLC, with an incidence of approximately 16.2-25% (16, 17). Especially since the new IAC pathological classification has been introduced, there are numerous reports that patients with a predominance of solid and micropapillary often present with poorer prognosis, have a pleural invasion, and present with intrapulmonary or lymphatic system metastases (18, 19). Bao et al. reported high N1 and N2 lymph node metastasis rates after systematic or selective lymph node dissection in patients with clinical T1aN0M0 NSCLC with micropapillary components (20). However, in these studies, micropapillary components (≥5%) were included in micropapillary predominant tumors, which may cause confounding effects on the role of NP-MCP in predicting lymphatic involvement risk in NCSLC. NP-MCP is a more common micropapillary pattern, and evaluation of its risk in OLNM is more valuable in guiding clinical decision making.

In our retrospective cohort, the clinical features of each patient were systematically analyzed; 717 (11.17%) patients were confirmed to have a micropapillary component, and OLNM was observed in 20.13% of patients with an NP-MCP of lung adenocarcinoma measuring ≤ 3 cm. Our data demonstrated that tumor size, young age of onset, and solid- and micropapillary-components were independent risk factors for OLNM, which is consistent with previous studies (21–25).

In terms of tumor markers, we report for the first time the diagnostic value of CYFRA21-1 in OLNM risk prediction (AUC=0.77, P < 0.001). Yoon et al. and Mei et al. reported that CYFRA 21-1 is a sensitive biomarker that can be used to detect lymph node metastasis in patients with breast cancer and esophageal squamous cancer (26, 27). The predictive value and biological mechanism of CYFRA21-1 in lymph node metastasis of lung adenocarcinoma need to be further confirmed in subsequent studies. Whether plasma CEA level could be an was a reliable risk indicator for ONM remains controversial. Wang et al. revealed that CEA >5.00 ng/ml was a risk indicator for OLNM, with a sensitivity and specificity of 68.32% and 78.55%, respectively (28). However, multivariate logistic regression analysis revealed that CEA level was not an OLNM risk factor in our research, which is consistent with the results of Zhang et al. (17). In addition, PET/CT was proclaimed to be useful in predicting OLNM in patients with early-stage T adenocarcinoma or squamous cell carcinoma (29), Matsushima et al. revealed that FDG SUVmax≥5 was a reliable risk predictor of lymph node metastasis upstage (30).

Lung adenocarcinoma, as the major subtype of NSCLC, consists of mixed pathological subtypes and not a single subtype. Thus, it is characterized by a wide range of molecular and pathophysiological variability. Based on morphological and molecular analyses, several clinicopathological features are reported to have association with patients’ prognosis, which has important implications for clinical treatment decisions (31). However, the genetic features of adenocarcinoma with NP-MCP have not been evaluated previously. Our research showed that EGFR exon alterations are quite common in NP-MCP patients, which was similar to previous reports in Chinese cohorts (32). Suda et al. reported a high EGFR mutation rate in multiple metastasis lung cancer patients with MP components (33). These studies also showed that amplification or overexpression of oncogenes could lead to a poorer prognosis. Kishi et al. reported that GEFR L858R RNA expression was detected more often in the micropapillary component than in other pathological subtype components, and they further indicated that adenocarcinoma patients with micropapillary component harboring L858R showed comparatively poor relapse free survival (8). In our study, exon 19 del mutation was found in 27.1% (44/162) of patients, and exon 21 L858R was detected in 26.0% (42/162) of the cases. And the remaining alteration types with frequencies less than 5.0% were exon 20 20-ins, exon 18 G719X, and exon 20 S768I, with frequencies of 1.9% (3/162), 4.3% (7/162), and 1.2% (2/162), respectively.

OLNM represents a different invasive extent than clinical lymph node metastasis owing to its specific clinicopathological features, which implies a unique prognosis and treatment of OLNM (34, 35). The biological mechanism and prognosis of OLNM require further exploration. To our knowledge, this is the first study to perform genomics analysis in patients with OLNM, and we found that EGFR exon 19 del (25, 56.9%) was the most frequent alteration, followed by EGFR exon 21 L858R (16, 36.4%) and other EGFR exon mutations (including exon 18 G719X, exon 20 20-ins, and exon 20 S768I; 6.8%). The analysis results revealed a significant association between EGFR exon 19 del alteration and OLNM (P < 0.001) compared with other EGFR exon mutations. Ou et al. indicated that the deletion of 19 exon might disrupt the EGFR kinase domain and improve its reactivity to EGFR-TKIs (36). Reviewing previous studies, we found that the mechanism of OLNM driven by 19 exon deletion remains unknown. The molecular biological mechanism of 19 del driving OLNM and its impact on the recurrence and prognosis of LA patients are the directions of our future research.

Despite previous studies, there is currently no prediction model for OLNM in patients with NP-MCP of lung adenocarcinoma in clinical practice, and optimal treatment guidelines for T1a-c stage patients are lacking. Our research analyzed the clinicopathological parameters related to the risk factors of OLNM through statistical analysis, then a reliable nomogram model (C-index = 0.84) was constructed to provide personalized risk predictions of OLNM. We hope this model will contribute to clinical decision making. For LA patients at high risk of OLNM, specific aggressive treatments, such as intensive systematic lymph node resection and postoperative chemotherapy, are recommended to reduce relapse and mortality rates (37–39).

There are certain limitations to our research. First, this was a retrospective research conducted at a single date center. Multicenter studies are required to validate these results. Second, Genetic mutation detection was performed according to the patients’ willingness. Thus, the sample size that had their genomics tested was a subset of the total cohort, making it challenging to include gene mutation information in multivariate regression analysis. Large-sample genomic detection is still needed to verify the role of gene alteration, especially rare gene mutations, in the patients with OLNM.

## Conclusions

ARMS-PCR suggested intense expression of EGFR exon 19 del characterizes lung adenocarcinoma in patients with NP-MCP and is a potential risk factor for OLNM. Caution is warranted in young patients. And high plasma CRFRA21-1 levels, large tumor size, and micropapillary/solid components are recommended for these patients because of the possibility of OLNM, aggressive lymph node dissection, and frequent follow-up. The molecular biological mechanism and prognostic value of exon 19 del in OLNM are new research prospects.

## Data Availability Statement

The original contributions presented in the study are included in the article/**Supplementary Material**. Further inquiries can be directed to the corresponding author.

## Ethics Statement

The studies involving human participants were reviewed and approved by Institutional Review Board of Qilu Hospital of Shandong University. The patients/participants provided their written informed consent to participate in this study.

## Author Contributions

Conceptualization, HT and KW. Methodology, YW. Software, KW. Validation, KW and RL. Formal analysis, CQ. Investigation, JQ and MX. Resources, JQ and MX. Data curation, LL and YW. Writing—original draft preparation, KW. Writing—review and editing, KW and YW. Visualization, KW and HT. Supervision, HT. Project administration, HT. All authors contributed to the article and approved the submitted version.

## Funding

This work was funded by National Key Research and Development Program (2021YFC2500904, and 2021YFC2500905) and Natural Science Foundation of Shandong Province (ZR2021LSW006).

## Conflict of Interest

The authors declare that the research was conducted in the absence of any commercial or financial relationships that could be construed as a potential conflict of interest.

## Publisher’s Note

All claims expressed in this article are solely those of the authors and do not necessarily represent those of their affiliated organizations, or those of the publisher, the editors and the reviewers. Any product that may be evaluated in this article, or claim that may be made by its manufacturer, is not guaranteed or endorsed by the publisher.

## Supplementary Material

Supplementary Figure 1 Immunohistochemistry validates the protein expression of the ARSM-PCR detected variants. Immunohistochemistry for EGFR **(A)**, KRAS **(B)**, ALK **(C)**, ROS1 **(D)**, RET **(E)**, and HER2 **(F)** protein expression (magnification ×200).
