# Supplementary figures and images for: Genomics Analysis and Nomogram Risk Prediction of Occult Lymph Node Metastasis in Non-Predominant Micropapillary Component of Lung Adenocarcinoma Measuring ≤ 3 cm
Source: Front Oncol. 2022 Jul 13;12:945997. doi: 10.3389/fonc.2022.945997 (PMC9326108; doi:10.3389/fonc.2022.945997)

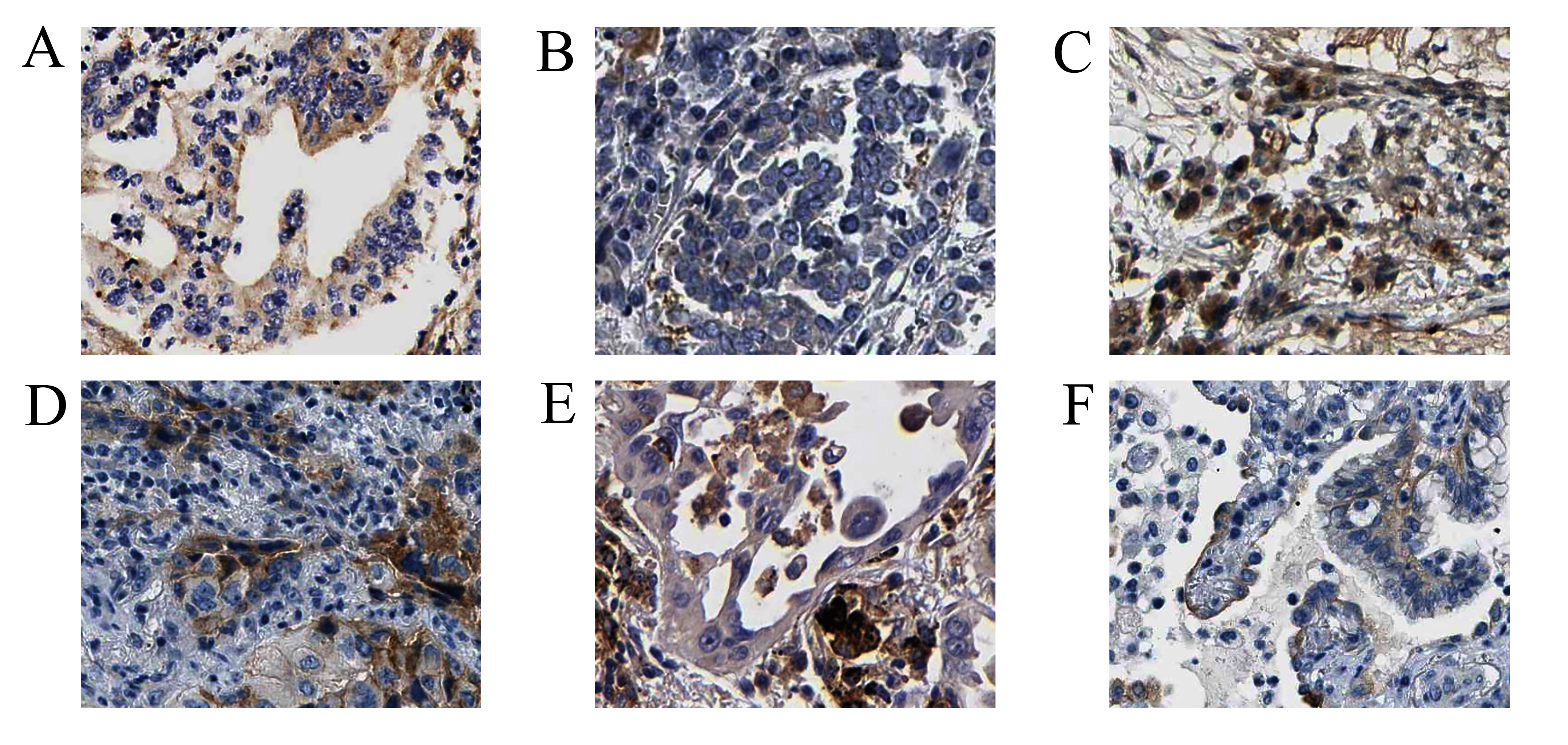

Supplement: Supplementary Figure 1 — Immunohistochemistry validates the protein expression of the ARSM-PCR detected variants. Immunohistochemistry for EGFR (A), KRAS (B), ALK (C), ROS1 (D), RET (E), and HER2 (F) protein expression (magnification ×200). [file Image_1.jpeg]
